# Integrative analysis of multi-dimensional imaging genomics data for Alzheimer's disease prediction

**DOI:** 10.3389/fnagi.2014.00260

**Published:** 2014-10-17

**Authors:** Ziming Zhang, Heng Huang, Dinggang Shen, The Alzheimer's Disease Neuroimaging Initiative

**Affiliations:** ^1^Department of Radiology and Biomedical Research Imaging Center, University of North Carolina at Chapel HillChapel Hill, NC, USA; ^2^Department of Computer Science and Engineering, University of Texas at ArlingtonArlington, TX, USA; ^3^Department of Brain and Cognitive Engineering, Korea UniversitySeoul, Republic of Korea

**Keywords:** Alzheimer's disease prediction, modality integration, imaging genomics data, feature selection, binary and multiclass classification

## Abstract

In this paper, we explore the effects of integrating multi-dimensional imaging genomics data for Alzheimer's disease (AD) prediction using machine learning approaches. Precisely, we compare our three recent proposed feature selection methods [i.e., multiple kernel learning (MKL), high-order graph matching based feature selection (HGM-FS), sparse multimodal learning (SMML)] using four widely-used modalities [i.e., magnetic resonance imaging (MRI), positron emission tomography (PET), cerebrospinal fluid (CSF), and genetic modality single-nucleotide polymorphism (SNP)]. This study demonstrates the performance of each method using these modalities individually or integratively, and may be valuable to clinical tests in practice. Our experimental results suggest that for AD prediction, in general, (1) in terms of accuracy, PET is the best modality; (2) Even though the discriminant power of genetic SNP features is weak, adding this modality to other modalities does help improve the classification accuracy; (3) HGM-FS works best among the three feature selection methods; (4) Some of the selected features are shared by all the feature selection methods, which may have high correlation with the disease. Using all the modalities on the Alzheimer's Disease Neuroimaging Initiative (ADNI) dataset, the best accuracies, described as (mean ± standard deviation)%, among the three methods are (76.2 ± 11.3)% for AD vs. MCI, (94.8 ± 7.3)% for AD vs. HC, (76.5 ± 11.1)% for MCI vs. HC, and (71.0 ± 8.4)% for AD vs. MCI vs. HC, respectively.

## 1. Introduction

Alzheimer's disease (AD) is a complex chronically progressive neurodegenerative disease and the most common form of dementia in elderly people worldwide. As reported in Wimo et al. (1997), the prevalence of clinically manifest AD is about 2% at the age of 65 years but increases to about 30% at the age of 85 years. Recent research (Brookmeyer et al., 2007) suggested the number of people with AD to be double within the next 20 years, and 1 in 85 people will be affected by 2050. With the increase of human's life expectancy, more and more elderly people will suffer from AD, and accordingly it will cause a heavy socioeconomic burden. Unfortunately, there is no treatment to cure or even slow the progression of this disorder currently (Weiner et al., 2012). Huge effort has been put on the better understanding of the disease for more effective treatment (Hardy and Selkoe, 2002; Jack et al., 2010; Weiner et al., 2010, 2012).

There are two more labels related to AD, that is, Healthy Control (HC) and Mild Cognitive Impairment (MCI) (Wee et al., 2011, 2012; Zhou et al., 2011; Zhang et al., 2012a). People in HC are actually not AD patients, while MCI can be considered as the early stage of AD (Dubois et al., 2010), in which people show mildly impaired in memory with relative preservation of other cognitive domains and functional activities and do not meet the criteria for dementia (Petersen et al., 2009). Also in Petersen et al. (2009) it was showed that each year 10–15% of MCI patients progressed to AD. As we see, the disease is developed gradually from HC to MCI, and eventually to AD. Between different statuses, there are no clear rules for defining the status of the disease. Therefore, accurate prediction of disease status (i.e., HC, MCI, or AD) becomes very difficult and important for early treatment of the disease.

To predict AD, a variety of biomarkers have been found and proposed (some of them are referred to by the sampling techniques), such as magnetic resonance imaging (MRI) Fan et al. (2007), positron emission tomography (PET), FDG-PET, and cerebrospinal fluid (CSF) (Hampel et al., 2008). Amount of works (Vemuri et al., 2009; Kohannim et al., 2010; Salas-Gonzalez et al., 2010; Hinrichs et al., 2011; Wolz et al., 2011; Zhang et al., 2011a; Ewers et al., 2012) have focused on how to utilize these biomarkers to classify AD, and they suggest that combining them for prediction is better than using any of them independently. Besides these existing biomarkers, recently genetic information is explored and studied in some very interesting works (Wang et al., 2012a,b; Nho et al., 2013a,b) for AD prediction.

In particular, machine learning community has provided powerful classification tools that are used for AD prediction (Zhang et al., 2011b, 2012b; Li et al., 2012; Liu et al., 2012; Zhang and Shen, 2012). Multiple kernel learning (MKL) framework (Rakotomamonjy et al., 2008; Zhang et al., 2010, 2011a) is one of the examples which can integrate different sources of information automatically using convex optimization. Feature selection (Liu et al., 2013) is another good example, where the first stage performs feature selection method, and in the second stage the selected features are fed into a classifier for training. In Wang et al. (2012a,b) the authors proposed group-sparse learning algorithms for regression and feature selection based on MRI, PET, and single-nucleotide polymorphism (SNP). The basic idea behind these algorithms is to select a small subset of features (i.e., feature selection) that will be commonly shared by different regression tasks.

The main contributions of this paper are two-fold:

**(1) Our first contribution is to compare the performances of three recent proposed feature selection methods from machine learning community in the same experimental environment**. These methods are multiple kernel learning (MKL) (Zhang et al., 2011a), high-order graph matching based feature selection (HGM-FS) (Liu et al., 2013), and sparse multimodel learning (SMML) (Wang et al., 2013). Feature selection for AD prediction has been attracting more and more attention. With very limited data samples and high dimensional data representations, it is reasonable to assume that all the data samples actually lie in a low dimensional representation space/manifold where classifiers can achieve better generalization, and feature selection is used to find such low dimensional manifold. However, in the literature such feature selection methods are developed independently using their own experimental settings, making it difficult to tell which is the best in terms of classification accuracy and how they behave on the data. Understanding these factors will be very useful for clinical usage to predict AD by choosing proper methods.

To answer such questions, in this paper we did comprehensive comparison between the three feature selection methods above on the Alzheimer's Disease Neuroimaging Initiative (ADNI)^1^ data set, which is designed to characterize clinical, genetic, imaging, and biochemical biomarkers of AD and identify the relationships between them over the course of disease progression from normal cognition to MCI to dementia. Using linear support vector machines (SVMs) as our classifiers, we report the classification accuracy, sensitivity, and specificity for both binary and multiclass classification tasks, based on 10-fold cross validation. Our experimental results suggests *(1) HGM-FS works best among the three feature selection methods, and (2) all the feature selection methods select some shared features*.

**(2) Our second contribution is to explore the effects of integrating multi-dimensional imaging genomics data for AD prediction based on the three feature selection methods above**. Developing discriminative features are always very important for AD prediction. Particularly, in this paper we focus on the performances of four widely-used modalities, namely MRI, PET, CSF, and SNP, with feature selection. This is very important and valuable for clinical purpose, because the cost of each modality is very different, and if the cheap ones can give us satisfactory results, we can utilize them first rather than utilizing costly modalities at the beginning. Our experimental results suggest that *in general, (1) PET is the best modality in terms of accuracy, and (2) adding SNP to other modalities does improve the classification accuracy, even though its discriminant power is weak*.

The rest of the paper is organized as follows. In Section 2 the materials used in our experiments are explained. In Section 3 the details of our classification methods are provided, including the preprocessing on genetic data, and our three recent proposed feature selection methods. In Section 4 our comparison results are listed and discussed. Finally, we conclude the paper in Section 5.

## 2. Materials

Our dataset is a subset from ADNI, where each subject can be represented using either imaging or genetic information. In total, we use 189 subjects from this dataset: 49 patients with AD, 93 patients with MCI, and 47 HC. Image preprocessing is performed separately for magnetic resonance imaging (MRI) and Fluorodeoxyglucose (FDG) Positron-Emission Tomography (PET) data. The preprocessing steps of MRI data include skull-stripping (Wang et al., 2011), dura removal, intensity inhomogeneity correction, cerebellum removal, spatial segmentation, and registration. We then parcellate the preprocessed images into 93 regions according to the template in Kabani et al. (1998). Only gray matter volume of these 93 regions-of-interest (ROI) is used in the experiments. For the preprocessing of PET images, we align the PET image of each subject to its corresponding MRI image using a rigid transformation and the average intensity of each ROI is calculated as a feature. CSF data were collected in the morning after an overnight fast using a 20- or 24-gauge spinal needle, frozen within 1 h of collection, and transported on dry ice to the ADNI Biomarker Core laboratory. In this study, CSF Aβ42, CSF *t*-tau, and CSF *p*-tau are used as features.

The single-nucleotide polymorphism (SNP) data (Saykin et al., 2010) were genotyped using the Human 610-Quad BeadChip. Among all SNPs, only SNPs, belonging to the top AD candidate genes listed on the AlzGene database (www.alzgene.org) as of June 10, 2010, were selected after the standard quality control (QC) and imputation steps. The QC criteria for the SNP data include (1) call rate check per subject and per SNP marker, (2) gender check, (3) sibling pair identification, (4) the Hardy-Weinberg equilibrium test, (5) marker removal by the minor allele frequency, and (6) population stratification. As the second pre-processing step, the quality-controlled SNPs were imputed using the MaCH software to estimate the missing genotypes. After that, the Illumina annotation information based on the Genome build 36.2 was used to select a subset of SNPs, belonging to the top 135 AD candidate genes (Bertram et al., 2007). The above procedure yielded 5677 SNPs from 135 genes. Because the dimensionality of SNPs is much higher than the ones of other neuroimaging features, we use the unsupervised feature selection method to reduce the dimensionality of SNPs to the similar level of other types of features.

## 3. Methodology

Our AD prediction framework is simple: Given the feature vectors for individuals, feature selection methods are applied first to select discriminative features. Then by performing element-wise product between the selected features and the learned weights by feature selection methods, the new feature vectors are fed into linear support vector machines (SVMs) to train the predictors. During testing, the selected features, learned weights, and predictors are fixed, and each test sample is classified into one of the three labels (i.e., AD, MCI, and HC) whose predicted score is the maximum.

In this section, we begin with introducing the preprocessing on genetic data, which attempts to reduce the computational time of each feature selection method beforehand by reducing the dimensionality of genetic data. Then we summarize our three recent proposed feature selection methods for readers to better understand our prediction framework.

### 3.1. Preprocessing on genetic data

Since the dimensionality of SNP features is so high that the computational time for the feature selection methods used in our experiments is very long, we perform a simple unsupervised dimension reduction method on the genetic data before applying those complicated feature selection in our experiments to reduce the computational time.

One commonly used unsupervised feature selection criterion is Laplacian Score (He et al., 2006), which aims to select features that can best preserve the local manifold structure. He et al. (2006) argued that: in many classification tasks, the local structure of data is expected to be more important than global structure.

Motivated by such observation, Laplacian Score was proposed to capture the local structure of data using graph Laplacian. Given a set of *N* training data {**x**_*i*}_*i*_ = 1, …, *N*_ where ∀*i*, **x**_*i*_ ϵ ℝ^*d*^ and the corresponding data matrix **X** ϵ ℝ^*d* × *N*^, a data similarity matrix **W** ϵ ℝ^*N* × *N*^ can be calculated using the heat kernel. By summing up the elements in **W** along each row, we can further create a diagonal matrix **D** ϵ ℝ^*N* × *N*^. The Laplacian Score of a feature is defined as $s(k)=\frac{X_{k}LX_{k}^{T}}{X_{k}DX_{k}^{T}}$, where **X**_*k*_ is the *k*th row in matrix **X** consisting of the values for the *k*th feature in all the data samples, **L** = **D** − **W** is the graph Laplacian matrix, and ( · )^*T*^ denotes the vector transpose operator.

One drawback of the Laplacian Score strategy is that: it is very sensitive to the heat kernel parameter σ which is used to construct similarity matrix. Thus, a huge amount of computation is needed to tune the parameter σ. On the other hand, constructing the similarity matrix itself is also time consuming [with the time complexity of at least 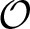
(*dN*^2^)].

The Laplacian score only considers the local structure. In order to utilize both local and global structures, we adopt an unsupervised local and global discriminative (LGD) feature selection criterion. The score of each feature is defined as the ratio between global variance and local variance:

(1) $$s(k)=\frac{\sum_{i}{\left(x_{i}(k)-\bar{x}(k)\right)}^{2}}{\sum_{j}\sum_{x_{i}(k)\in o(x_{j}(k))}{\left(x_{i}(k)-{\bar{x}}_{j}(k)\right)}^{2}},$$

where **x**_*i*_(*k*) is the value for the *k*th feature of the *i*th sample, **x**(*k*) is the mean value for the *k*th feature in the data samples, *o*(**x**_*j*_(*k*)) is the set of neighbor points of the *j*th sample. **x**_*j*_(*k*) is the mean computed from the set of neighbor points as ${\bar{x}}_{j}(k)=\frac{\sum_{x_{i}(k)\;\in \;o(x_{j}(k))}x_{i}(k)}{\left|o(x_{j}(k))\right|}$, where |*o* (**x**_*j*_(*k*))| is the cardinality of the neighbor set. Because the neighbor points of point **x**_*i*_(*k*) can be efficiently computed by sorting the values {**x**_*i*_(*k*)}_*i* = 1, …, *N*_, the computational complexity of our method is 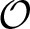
(*dN* log *N*).

### 3.2. Feature selection methods

In general, feature selection methods can be considered as the combination of (1) data fitting terms and (2) sparse-induced regularization terms. Data fitting terms guarantee that the learned models are suitable for prediction within certain variations, while sparse-induced regularization terms make sure that only the “important” features have non-zero weights. By designing different combinations of these two terms, different feature selection methods are developed. For instance, hinge-loss or least-square loss can be used as data fitting terms, and ℓ_1_ norm, trace norm, or group sparsity can be used as sparse-induced regularization terms. Typically, different combinations result in selecting different features, and we briefly summarize the details of MKL, HGM-FS, and SMML to show how they developed for feature selection.

#### 3.2.1. Multiple kernel learning (MKL)

The basic idea of MKL is to build an optimal kernel for a specific task (e.g., classification) based on a set of basis kernels. In our experiments, we employ SimpleMKL (Rakotomamonjy et al., 2008) as the MKL competitor. Given training samples {(**x**_*i*_,*y*_*i*_)}_*i* = 1, …, *N*_, where ∀*i*, **x**_*i*_ ϵ ℝ^*d*^ is an input data vector and *y*_*i*_ ϵ {1, −1} is its binary label, and *M* feature mapping functions {ϕ_*m*_ : ℝ^*d*^ → ℝ^*D*_*m*_^}_*m* = 1, …, *M*_, SimpleMKL formulates the MKL problem with the hinge loss function for binary classification as follows:

(2) $$\begin{array}{l} \underset{{}_{\beta ,w,b}}{\min}\sum_{m}\frac{\|w_{m}{\|}_{2}^{2}}{2\beta_{m}}+C\;\sum_{i}\ell (x_{i},y_{i};w,b)\\ \text{s.t. }\forall m,\beta_{m}\geq 0,\;\|\beta {\|}_{1}\leq 1 \end{array}$$

where ∀*m*, β_*m*_ denotes the weight for kernel *m* induced by feature mapping function ϕ_*m*_, β denotes the kernel weight vector, **w** = {**w**_*m*_} and *b* denote the classifier parameters, *C* ≥ 0 is a predefined regularization parameter, ‖ · ‖_1_ denotes the ℓ_1_ norm of a vector, ∀*i*, ℓ(**x**_*i*_, *y*_*i*_; **w**, *b*) = max {0, 1 − *y*_*i*_ [∑_*m*_ **w**^*T*^_*m*_ϕ_*m*_(**x**_*i*_) + *b*]} denotes the hinge loss function, which is used in all the optimization problems in this paper, and ( · )^*T*^ in ℓ denotes the matrix transpose operator. We adopt the method in Xu et al. (2010) to solve MKL in Equation 2.

#### 3.2.2. High-order graph matching based feature selection (HGM-FS)

(Liu et al., 2013) This method extends the traditional LASSO (Tibshirani, 1994) by adding two regularizers which capture the geometrical relations (i.e., high-order statistics) between the predicted vectors and the target vectors (e.g., class label vectors). The underlying assumption of the method is that the predicted vectors are not only close to the target vectors in the target space, but also distributed similarly to the target vectors.

Given a set of training data {**x**_*i*_, **y**_*i*_}_*i* = 1, …, *N*_ where ∀*i*, **x**_*i*_ ϵ ℝ^*d*^ is a *d*-dimensional feature vector, and **y**_*i*_ ϵ ℝ^
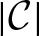
^ is its associated 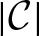-dimensional target vector, we denote **X** = [**x**_1_, …, **x**_*N*_] ϵ ℝ^*d* × *N*^ as the feature matrix, and **Y** = [**y**_1_, …, **y**_*N*_] ϵ ℝ^
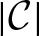
×*N*^ as the target matrix. Letting **W** ϵ ℝ^*d* × 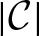
^ be the regression coefficient matrix, and ‖ · ‖_*F*_ denote the Frobenius norm, the two new regularizers are defined as $B=\sum_{i,j\;=\;1}^{N}\|(y_{i}-y_{j})-W^{T}(x_{i}-x_{j}){\|}_{F}^{2}$ and $T=\sum_{i,j,K\;=\;1}^{N}\|{\left(y_{i}-y_{j}\right)}^{T}(y_{j}-y_{k})-{\left(x_{i}-x_{j}\right)}^{T}WW^{T}(x_{j}-x_{k}){\|}_{F}^{2}$, where *B* and *T* capture the pair-wise and triplet-wise geometrical relations in the target space between the predicted vectors and the target vectors, respectively. Therefore, the final optimization formulation for HGM-FS can be written down as follows:

(3) $$L(W)=\underset{W}{\min}\|W^{T}X-Y{\|}_{F}^{2}+\lambda_{1}\|W{\|}_{1}+\lambda_{2}B+\lambda_{3}T$$

where λ_1_ ≥ 0, λ_2_ ≥ 0, and λ_3_ ≥ 0 are regularization parameters. In theory, it is possible to add any higher-order graph matching information into the objective function above, and the features with non-zero regression coefficients from the original feature space are selected for final classification.

#### 3.2.3. Sparse multimodel learning (SMML)

(Wang et al., 2013) SMML was proposed to integrate heterogeneous features from different modalities by using the joint structured sparsity regularizations to learn the feature importance from both group-wise and individual point of views.

Let {**x**_*i*_, **y**_*i*_}_*i* = 1, …, *N*_ be *N* training samples, where each input vector ∀*i*, **x**_*i*_ = {(**x**^1^_*i*_)^*T*^, …, (**x**^*K*^_*i*_)^*T*^} ϵ ℝ^*d*^ has $d=\sum_{j\;=\;1}^{K}d_{j}$ dimensions containing all features from *K* modalities in total, each modality *j* has *d*_*j*_ dimensional feature vector, **y**_*i*_ ϵ ℝ^
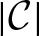
^ is its associated 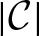 dimensional class label vector, and 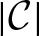 is the number of classes. Let **X** = [**x**_1_, …, **x**_*N*_] ϵ ℝ^*d* × *N*^ and **Y** = [**y**_1_, …, **y**_*N*_] ϵ ℝ^
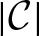
×*N*^. We denote the classification coefficient matrix as **W** = [**w**^1^_1_, …, **w**^1^_
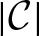
_; … ;**w**^*K*^_1_, …, **w**^*K*^_
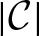
_] ϵ ℝ^*d* × 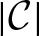
^, where **w**^*q*^_*p*_ ϵ ℝ^*d*_*q*_^ is the weights of all features from the *q*th modality in the classification decision function of the *p*th class.

Based on these notations, SMML can be formulated as follows: 
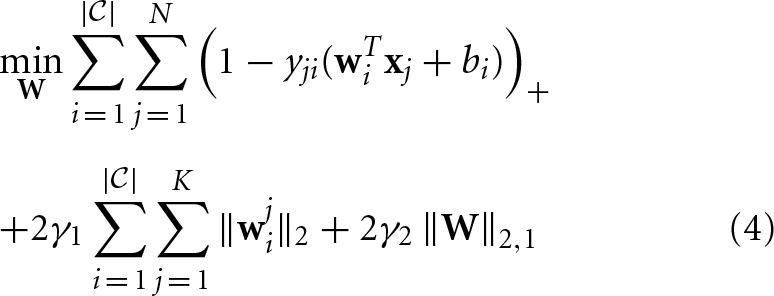
 where *y*_*ji*_ ϵ {−1, 1} denotes the binary class label for data **x**_*i*_, **w**_*i*_ ϵ ℝ^*d*^ denotes the classification coefficient vector for class *i*, **w**^*j*^_*i*_ ϵ ℝ^*d*_*j*_^ denotes the classification coefficient vector for class *i* and modality *j*, γ_1_ ≥ 0 and γ_2_ ≥ 0 are two predefined regularization parameters, ( · )_+_ = max{0, ·}, and ‖ · ‖_2,1_ denotes the ℓ_2, 1_ norm of a matrix. Both the regularizers try to capture the essential structure in the classification coefficient matrix to improve the performance. In Wang et al. (2013), a very efficient algorithm has been proposed to solve Equation 4, which is guaranteed to converge to a global optimal solution.

## 4. Results

In order to verify the effect of integration of both imaging and genetic information on the AD prediction accuracy, we perform two classification tasks separately: (1) binary classification, i.e., AD vs. MCI, AD vs. HC, and MCI vs. HC; (2) multiclass classification, i.e., AD vs. MCI vs. HC. Ten-fold cross validation is utilized for evaluating each method. LIBSVM (Chang and Lin, 2011) is employed as our linear SVM solver. The parameters for each method is determined using grid search. Best performance of each method is reported.

### 4.1. Data process

For MRI, PET, and CSF, we utilize all the features, that is, 93 + 93 + 3 = 189 in total. For genetic SNP data, we apply the preprocessing method to rank the 5677 SNPs in a descending order, and finally preserve the top 189 SNPs as the final features for the genetic data without fully tuning, because we would like to make the data from both imaging and genetic information sources balanced.

For MRI, PET, and CSF, each feature is normalized by subtracting the mean and then divided by the standard deviation, where the mean and the standard deviation are calculated from training data. That is, given a set of training data {**x**_*i*_}_*i* = 1, …, *N*_, every single data sample $\tilde{x}$ needs to be normalized as $\forall j,\tilde{x}(j)\leftarrow \frac{\tilde{x}(j)-\bar{x}(j)}{\sqrt{\frac{1}{N}\sum_{i\;=\;1}^{N}{\left[x_{i}(j)-\bar{x}(j)\right]}^{2}+\epsilon }}$, where $\bar{x}=\frac{1}{N}\sum_{i\;=\;1}^{N}x_{i}$ denotes the mean of the training data, *j* denotes the *j*th feature in each feature vector, and ϵ is a very small positive constant to avoid the case of the dominator equal to zero.

For genetic SNP features, each feature is normalized by subtracting the minimum value along the dimension and then divided by the difference between the maximum and the minimum along the dimension as well, calculated from training data. This normalization process can be written as $\forall j,\tilde{x}(j)\leftarrow \frac{\tilde{x}(j)-\min_{i\;=\;1,\cdots ,N}x_{i}(j)}{\max_{i\;=\;1,\cdots ,N}x_{i}(j)-\min_{i\;=\;1,\cdots ,N}x_{i}(j)+\epsilon }$.

To generate the *final feature vectors* as the input for each feature selection method, each feature is further normalized to the ℓ_2_-norm unit ball based on the training data. This normalization can avoid the scaling bias in each feature. Again, this normalization process can be written as $\forall j,\tilde{x}(j)\leftarrow \frac{\tilde{x}(j)}{\sqrt{\sum_{i\;=\;1}^{N}x_{i}{\left(j\right)}^{2}+\epsilon }}$.

### 4.2. Binary classification

In this experiment, we perform three binary classification tasks: AD vs. MCI, AD vs. HC, and MCI vs. HC, respectively. For each task, the former is the positive class, and latter is the negative class. We also test 9 different configurations of modalities as features for feature selection methods: (1) MRI only, (2) PET only, (3) CSF only, (4) SNP only, (5) MRI+SNP, (6) PET+SNP, (7) CSF+SNP, (8) MRI+PET+CSF, and (9) MRI+PET+CSF+SNP. Linear SVMs with the final features are utilized as the baseline method, because there is no feature selection involved.

**Figure 1:**
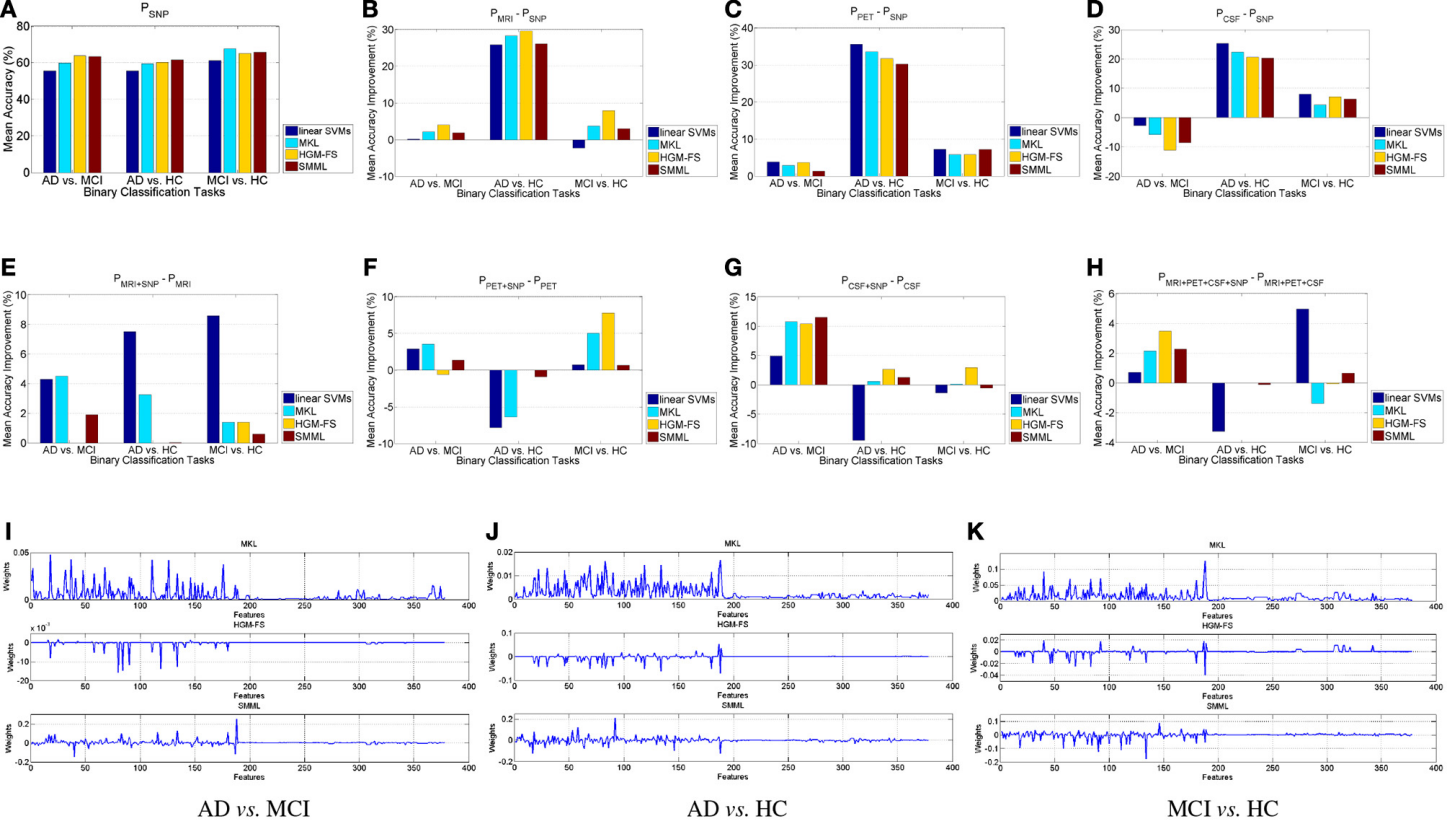
**Binary classification**. **(A)** Classification performance using SNP features. **(B–D)** Mean accuracy percentage improvement using MRI, PET, CSF, respectively, compared to using SNP, with the four different classification methods, compared to the performance using SNP. **(E–H)** Mean accuracy percentage improvement, by adding SNP features, using different classification methods. **(I–K)** Learned weights by MKL, HGM-FS, and SMML, respectively, using all the four feature modalities, whose order is 93-dim MRI, 93-dim PET, 3-dim CSF, and 189-dim SNP. The weights shown in the figure are average over the 10-fold cross validation.

We first analyze the performance using each individual modality, compared to the performance using SNP. Figure 1A shows the performance using SNP features, and Figures 1B–D show our comparison results. From Figure 1A we can see that using SNP feature selection based classifiers outperform the baseline method consistently, but among them there is no winner for all the binary classification tasks, and their performances are similar, in general. From Figures 1B–D we can see that, overall, MRI and PET outperform SNP significantly for all the methods and all the binary classification tasks, especially for AD vs. HC. For CSF, only for AD vs. MCI, its performances of all the methods are worse than those SNP correspondingly. For the other two classification tasks, the performances are still much better than using SNP. Notice that the dimensionality of CSF features is only 3, which leaves little room for selecting important features, while the dimensionality of SNP features are 189. However, it seems that the discriminative power of SNP features are very weak, which results in that even the dimensionality of the features is higher, its performance is still worse than others. General speaking, for AD prediction on the binary classification tasks, the discriminative power of different modalities can be ordered as PET>MRI>CSF>SNP.

Next we compare the performance with/without SNP features using MRI, PET, CSF, respectively, and MRI+PET+CSF. Figures 1E–H shows our comparison results. For AD vs. HC, by adding the SNP features the performance using each method improves little, in general, especially for each feature selection based classifiers. This is mainly because the performance using the rest modalities without SNP are almost saturated, and under this circumstance the SNP features will be considered as noisy features, which has little contribution to the performance. Another reason is that AD vs. HC is the easiest task due to their differences. For the other two binary classification tasks, especially for AD vs. MCI, the performance improvement by adding SNP features is significant for all the methods. This is mainly because the disease statuses in each task have no clear differentiation, which makes the discriminative power of the rest modalities quite weak as well, and in this case the SNP features can provide complementary information to help differentiate the disease statuses. Overall, even though the discriminative power of SNP features for AD prediction are rather weak, these features do help other modalities improve the performance for each feature selection based classifier, especially on AD vs. MCI.

Table 1 shows the classification performances of different methods using all the four modalities. As we see, all the feature selection based classifiers outperform the baseline method significantly, but still there is no winner for all the binary classification tasks. Among the three feature selection based classifiers, HGM-FS works the best, which has achieved the state-of-the-art performance.

**Table 1:**
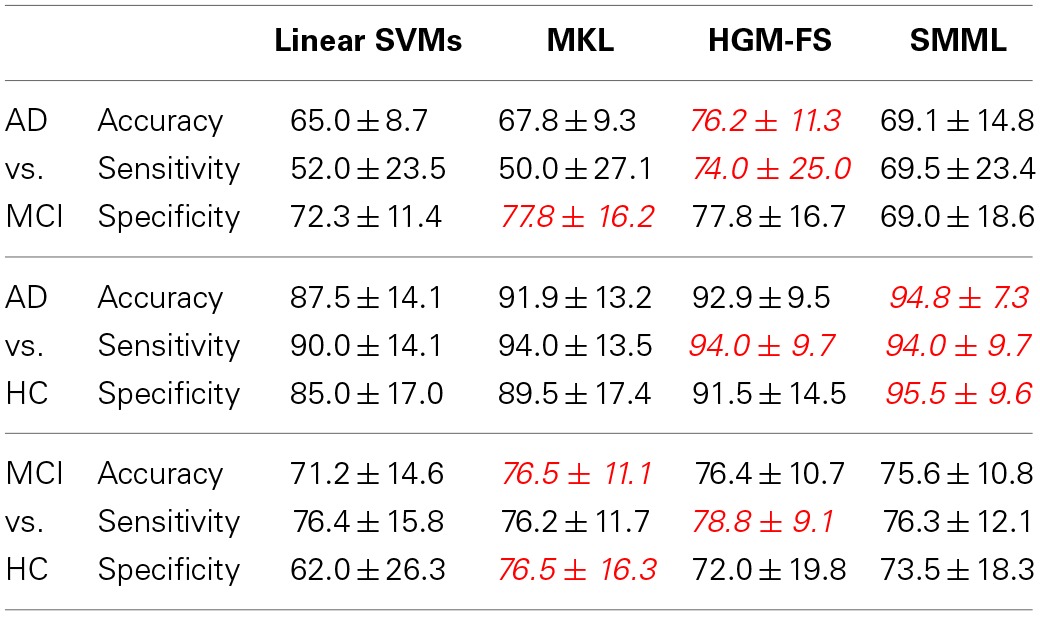
**Performance comparison (%) among different methods using all the four modalities (i.e., MRI, PET, CSF, and SNP) in terms of “mean ± standard deviation” for binary classification tasks (i.e., positive class vs. negative class)**.

*For better comparison, we highlight the best performances using red color*.

Finally we analyze the learned weights by each feature selection method using all the four modalities, as shown in Figures 1I–K. As we see, the weights for SNP features are relatively smaller than those for the other modalities, on average, and most of them are zeros. This observation demonstrates that the discriminative power of SNP features is much weaker than that of the other three modalities, which is consistent with the observation in Figure 1. However, these small non-zero weights are still very important for improving the performance for AD prediction. Also, for these methods, some of the selected features are shared, which may be very important for the prediction of the disease status. Among the three methods, the selected features by HGM-FS are sparsest for every binary classification task.

### 4.3. Multiclass classification

For multiclass classification, we conduct three “one-vs-the-rest” binary classification tasks instead, i.e., HC vs. non-HC, MCI vs. non-MCI, and AD vs. non-AD. Then a test data sample is classified to the class whose decision value is the maximum among the three binary classification tasks. Notice that multiclass classification is more difficult than binary classification in general.

**Figure 2:**
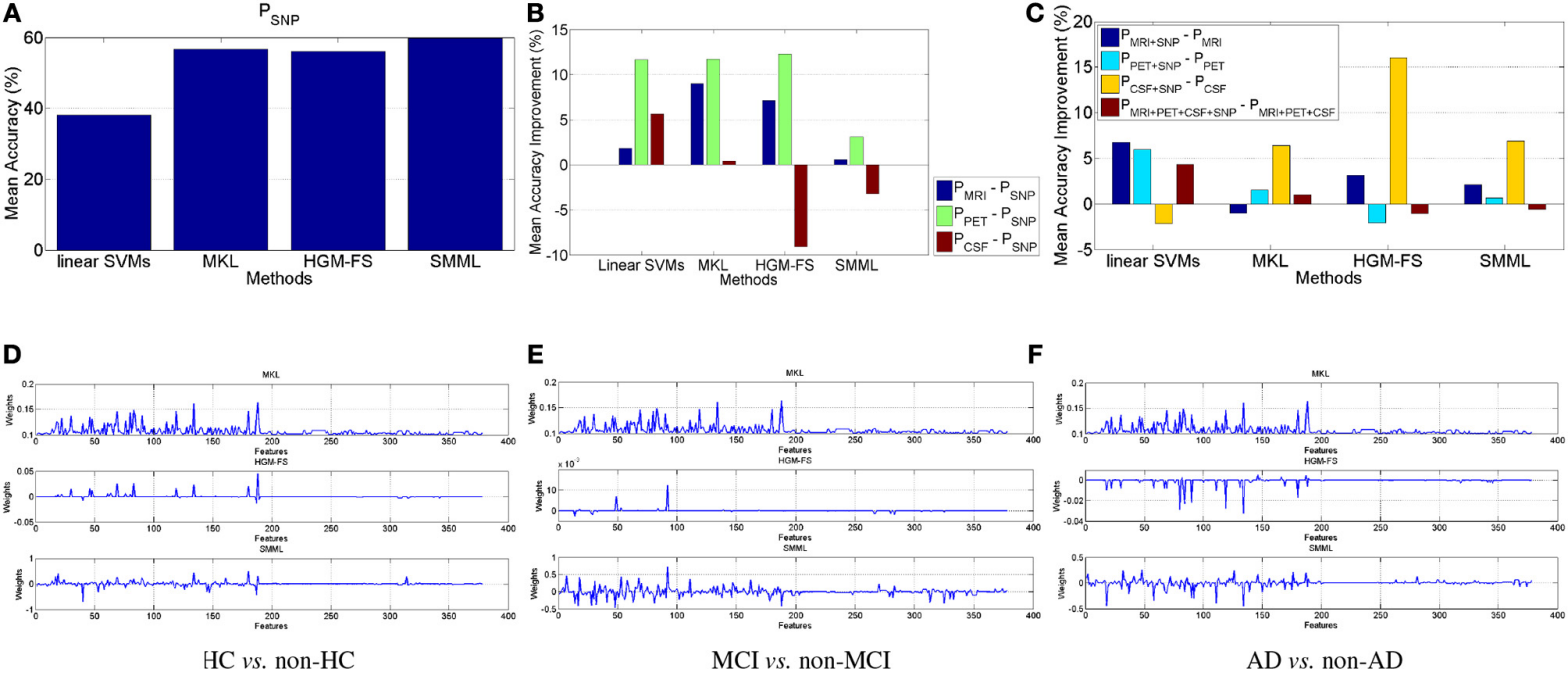
**Multiclass classification**. **(A)** Classification performance of the four methods using SNP features. **(B,C)** Performance improvement in terms of mean accuracy using the four different methods **(B)** by comparing performance of MRI, PET, CSF, respectively, with that of SNP, and **(C)** by comparing different modalities with/without genetic SNP features. **(D–F)** Learned weights by MKL, HGM-FS, and SMML, respectively, for multiclass classification using all the four feature modalities, whose order is 93-dim MRI, 93-dim PET, 3-dim CSF, and 189-dim SNP. The weights shown in the figure are average over the 10-fold cross validation. For MKL, its learned weights keep unchanged among the three “one-vs-the-rest” binary classification tasks.

As we did for the binary classification, we perform similar analysis for the multiclass classification as well. Figure 2A shows the performance of each method using SNP features, which is slightly worse than the corresponding performance in Figures 1A and 2B shows the performance improvement using MRI, PET, CSF, respectively, compared to the performance using SNP. Still MRI and PET work better than SNP. However, different from binary classification above, CSF works worse than SNP, especially for feature selection based classifiers. This is because that the much lower dimensionality of CSF features makes it much more difficult to distinguish multiple classes simultaneously. To summarize, for AD prediction using multiclass classification, MRI and PET are better than both CSF and SNP, and PET again works best.

Figure 2C shows the performance improvements for all the four methods by comparing the performances using different modalities with/without SNP. Similar to Figures 1E–H, in general, adding SNP features can improve multiclass classification performance. And Table 2 lists the performances of the four methods using all the modalities. Still HGM-FS outperforms the rest methods, and has achieved the state-of-the-art result on AD prediction using multiclass classification.

**Table 2:**
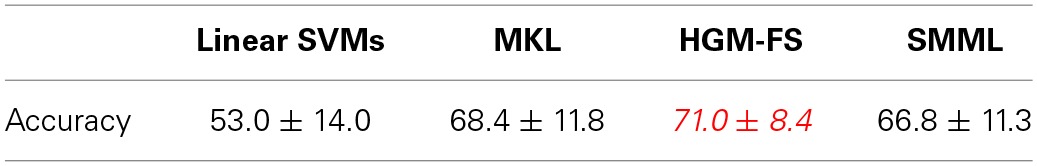
**Performance comparison (%) among different methods using all the four modalities (i.e., MRI, PET, CSF, and SNP) in terms of “mean ± standard deviation” for the multiclass classification task (i.e., AD vs. MCI vs. HC)**.

*For better comparison, we highlight the best performances using red color*.

Figures 2D–F shows the learned weights by MKL, HGM-FS, and SMML, respectively, for the three “one-vs-the-rest” binary classification tasks using all the four modalities. Again, on each binary classification task, the learned weights for SNP features are relatively smaller than those for the rest modalities, some of the features with non-zero weights are shared as well, and among the three methods, HGM-FS produces the sparsest selected features for multiclass classification.

### 4.4. Top selected genetic basis

**Figure 3:**
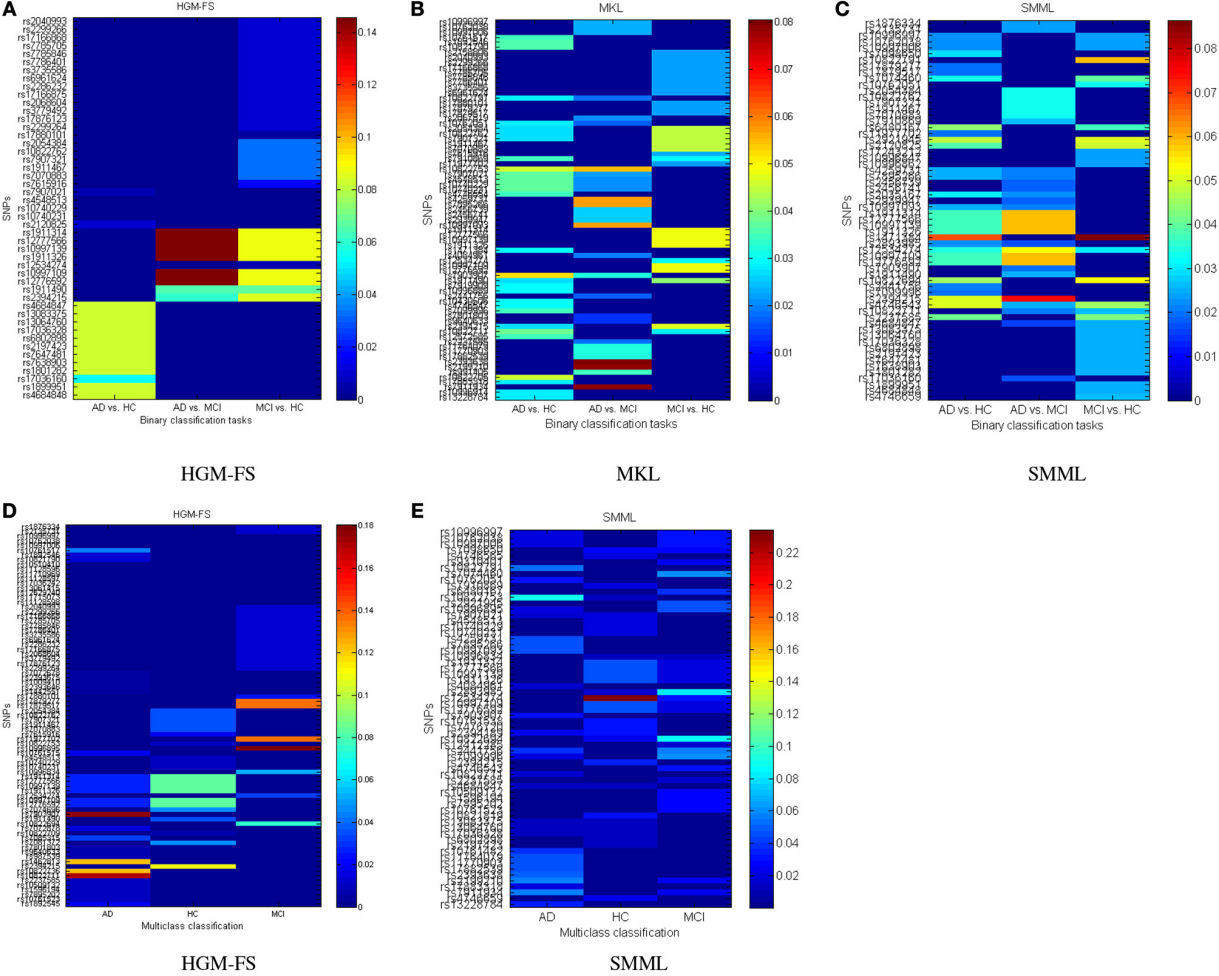
**Top selected SNPs using different methods for **(A–C)** binary classifications, and **(D,E)** multiclass classification**. The MKL method has the identical results in binary and multiclass classifications as shown in **(B)**. The color map shows the learned feature weights in classifications.

The top selected SNPs by three different methods in binary classifications are plotted in Figure 3A (HGM-FS method), Figure 3B (MKL method), and Figure 3C (SMML method). In all figures, the color map is used to show the weights of different features in classifications. If the weight is large, the feature is important to the corresponding class.

The top selected SNPs by two different methods in multiclass classifications are plotted in Figure 3D (HGM-FS method) and Figure 3E (SMML method). The MKL method has the identical results in binary and multiclass classifications, thus the selected SNPs by MKL method are the same as Figure 3B.

Because the feature selection mechanisms are different in three methods, the top selected SNPs are different. However, after our careful investigations, we found that many of the most top selected SNPs among three methods come from the same several genes. For example, the most top selected SNPs in HGM-FS method in multiclass classifications come from gene “CTNNA3,” which is a protein-coding gene and is associated to late-onset Alzheimer's disease (Miyashita et al., 2007). The most top selected SNPs in SMML method in multiclass classifications come from gene “PON2,” which encodes paraoxonase-2 gene and is associated with apolipoprotein E4 allele in both Alzheimer's and vascular dementias (Janka et al., 2001). The most top selected SNPs in MKL method in multiclass classifications also come from gene “CTNNA3” and gene “PON2.” Although the well-known APOE SNP is not the top one SNP in our list, it still appears in the top rank list. Because our studies are data-driven integrative multi-variate studies, our results are consistent with the existing GWAS results but also show the difference. The top selected SNPs in our studies reveal more interactions between genotypes and phenotypes.

## 5. Discussion and conclusions

In this paper, we conduct a comprehensive study on modality integration for Alzheimer's disease (AD) prediction using the ADNI dataset. We employ four widely-used modalities (i.e., MRI, PET, CSF, and SNP), and compare three state-of-the-art feature selection based linear classifiers (i.e., MKL/HGM-FS/SMML + linear SVMs) with the baseline classifier (i.e., linear SVMs without feature selection). In our experiments, we perform both binary classification (i.e., AD vs. MCI, AD vs. HC, and MCI vs. HC) and multiclass classification (i.e., AD vs. MCI vs. HC), and analyze the results, respectively, based on 10-fold cross validation.

The key observations from our experimental results are: (1) Among all the compared methods, MRI and PET perform better than CSF and SNP in terms of prediction accuracy, and PET is the best among all the four modalities; (2) In general, SNP performs worst, but it is still helpful to improve the performance with other modalities together; (3) Among the three feature selection based classifiers, HGM-FS with linear SVMs performs best, and using all the four modalities, it has achieved the state-of-the-art performance for either binary or multiclass classification for AD prediction; (4) The selected features by each method share some common parts, the learned weights for SNP features are relatively smaller than those for the others, and HGM-FS produces the sparsest features among the three methods.

Our results are also very useful for clinical usage. For instance, considering the prediction performance, PET should be preferred, which is consistent with some recent evidences in the diagnosis of AD (Sperling and Johnson, 2013). For AD vs. HC, imaging modalities are highly recommended, and adding genomics data will, generally speaking, harm the classification accuracy. However, for AD vs. MCI, considering all the imaging and genomics data is highly recommended, since these modalities contain complementary information and integration of these information for diagnosis will be very helpful. Among the compared feature selection methods, one should be chosen according to the classification task, which will maximize the accuracy, e.g., for AD vs. HC, SMML is preferred rather than HGM-FS. Several imaging and genetic features are commonly selected by the feature selection methods in our experiments. These features may have high correlation with AD, and understanding why and how these features change may provide useful evidence to understand AD.

### Conflict of interest statement

The authors declare that the research was conducted in the absence of any commercial or financial relationships that could be construed as a potential conflict of interest.

## Footnotes

^1^This dataset can be downloaded from http://www.loni.ucla.edu/ADNI.
